# Locating potentially lethal genes using the abnormal distributions of genotypes

**DOI:** 10.1038/s41598-019-47076-w

**Published:** 2019-07-22

**Authors:** Xiaojun Ding, Xiaoshu Zhu

**Affiliations:** School of Computer Science and Engineering, Yulin Normal University, Yulin, 537000 China

**Keywords:** Computational biology and bioinformatics, Computational models, Statistical methods

## Abstract

Genes are the basic functional units of heredity. Differences in genes can lead to various congenital physical conditions. One kind of these differences is caused by genetic variations named single nucleotide polymorphisms (SNPs). An SNP is a variation in a single nucleotide that occurs at a specific position in the genome. Some SNPs can affect splice sites and protein structures and cause gene abnormalities. SNPs on paired chromosomes may lead to fatal diseases so that a fertilized embryo cannot develop into a normal fetus or the people born with these abnormalities die in childhood. The distributions of genotypes on these SNP sites are different from those on other sites. Based on this idea, we present a novel statistical method to detect the abnormal distributions of genotypes and locate the potentially lethal genes. The test was performed on HapMap data and 74 suspicious SNPs were found. Ten SNP maps “reviewed” genes in the NCBI database. Among them, 5 genes were related to fatal childhood diseases or embryonic development, 1 gene can cause spermatogenic failure, and the other 4 genes were associated with many genetic diseases. The results validated our method. The method is very simple and is guaranteed by a statistical test. It is an inexpensive way to discover potentially lethal genes and the mutation sites. The mined genes deserve further study.

## Introduction

Genes are the most important genetic materials that determine the health of a person. The functions of genes may be affected by genetic variations called single nucleotide polymorphisms (SNPs), so it is important to study disease-related genes from SNPs. There are many defective genes caused by SNPs that result in human Mendelian diseases (i.e., single gene diseases)^1,2^. For example, Prescott *et al*.^3^ found a nonsynonymous SNP in ATG16L1 related to Crohn’s disease. Seki *et al*.^4^ reported that a functional SNP in CILP is potentially linked to lumbar disc disease. These discoveries have inspired researchers to continue their investigations into SNPs. However, the known pathogenic genes caused by SNPs comprise only a small fraction of the information we have about diseases; many of the gentic problems caused by SNPs are still unknown. The number of SNPs is very large and most SNPs do not seem to have effects on genes^5–7^. Evaluating every SNP with experiments is expensive, but narrowing the range of potentially dangerous SNPs will benefit the study of pathogenic genes^8^. Researchers have analyzed SNPs from various angles. Lee *et al*.^9^ built a functional SNP database that integrates information from 16 bioinformatics tools and the functional SNP effects from disease research. Cargill *et al*.^10^ studied the different rates of polymorphism within genes and between genes. They concluded that the rates may reflect selection acting against deleterious alleles during evolution and the lower allele frequency of missense cSNPs (coding-region SNPs) are possibly associated with diseases. Adzhubei *et al*.^11^ developed a tool named PolyPhen, which predicts the possible impact of an amino acid substitution on the structure and function of a human protein. Kumar *et al*.^12^ developed a tool named SIFT that predicts whether an amino acid substitution will affect protein function. Their algorithm is sensitive to naturally-occurring nonsynonymous polymorphisms and laboratory-induced missense mutations. However, synonymous mutations can also contribute to human diseases^13^. For example, Westerveld *et al*.^14^ reported that the intronic variant rs1552726 may affect the splice site activity.

Here, we propose a novel method based on genetic laws. A defective gene caused by SNPs can lead to fatal diseases that prevent fertilized embryos from developing into normal fetuses or the sufferers from these defective genes die in childhood. These defective genes affect the distributions of genotypes on the SNPs. This approach provides a novel way to distinguish the pathogenic SNPs from normal SNPs.

## Results

In our experiments, the statistical test was checked on each chromosome. The common bi-allele SNPs in the 11 populations were extracted for 1 chromosome. On chromosome 1, there were 117,068 common SNPs and 8 SNPs were suspicious. On all 22 chromosomes, 74 SNPs were selected from among the 1,395,560 SNPs (we did not check SNPs on the X and Y chromosomes because the heredity characteristics from the X and Y chromosomes are different than those on autosomes). We looked up the suspicious SNPs in the NCBI database, and located 10 “reviewed” genes. A “reviewed” gene means that its RefSeq record has been reviewed by NCBI staff or by a collaborator. The NCBI review process includes assessing the available sequence data and the literature. Some RefSeq records may incorporate expanded sequence and annotation information. The corresponding chromosomes, SNPs, genes, gene types, alleles, disease patterns, and p-values are listed in Table 1. For the first 2 SNPs and their genotypes, the expectations of the number of individuals in each population are listed in Tables 2 and 3. For the genotype ‘AA’ at SNP site rs2145402, there should be about 34.4 individuals in all populations. However, none were observed. If genotype ‘AA’ is normal, the probability of the event is only 3.05E-16, which means that the event has too small of a probability to happen by chance. We speculated that the distribution of the genotypes at SNP rs2145402 is abnormal. The corresponding gene LYST can potentially result in genetic diseases.

**Table 1:** Information of SNPs mapping “reviewed” genes.

Chr	SNP	Gene	Alleles	Suspicious Pathogenic Genotype	P Value
1	rs2145402	LYST	A/C	AA	3.05E-16
1	rs4915931	ROR1	A/G	AA	1.70E-12
1	rs4660992	BMP8B	C/T	TT	9.91E-10
6	rs9263745	CCHCR1	A/G	AA	5.67E-11
7	rs11766679	DPP6	A/G	GG	4.29E-10
10	rs12263497	INPP5F	A/G	GG	1.46E-10
11	rs1552726	NLRP14	A/G	GG	2.64E-09
14	rs3742943	JAG2	C/T	TT	6.07E-08
16	rs1646233	CBFA2T3	A/G	AA	1.20E-09
22	rs11705619	TXNRD2	C/T	CC	1.43E-09

**Table 2:** Expectations of the number of individuals in each population who have genotype ‘AA’ at SNP site rs2145402.

Population	CHB	CHD	JPT	CEU	TSI	ASW	LWK	MKK	YRI	GIH	MEX	All
Expectation	1.1	0.4	0.5	11.6	8.2	1.0	0.0	0.1	0.1	4.8	6.6	34.4

**Table 3:** Expectations of the number of individuals in each population who have genotype ‘AA’ at SNP site rs4915931.

Population	CHB	CHD	JPT	CEU	TSI	ASW	LWK	MKK	YRI	GIH	MEX	All
Expectation	0.2	0.1	0.2	8.8	4.2	2.5	1.3	0.3	2.5	4.1	2.5	26.7

We discuss the related studies from the literature in the following section. We found that most of these genes are actually associated with fatal genetic diseases.

1. SNP rs2145402 maps gene LYST
  In the ClinVar database^15^, LYST is associated with lung cancer, malignant melanoma, and Chediak-Higashi syndrome. Many researchers^16–21^ have reported that the gene LYST is associated with Chediak-Higashi syndrome, which can affect many parts of the body, particularly the immune system. The disease damages immune system cells. Most affected individuals have repeated and persistent infections starting in infancy or early childhood^22^. The result of the disease is very serious and most affected individuals die in childhood^23^.
2. SNP rs4915931 maps gene ROR1
  In the ClinVar database^15^, ROR1 is associated with malignant melanoma. Broome *et al*.^24^ reported that ROR1 is a receptor tyrosine kinase expressed during embryogenesis, chronic lymphocytic leukemia, and in other malignancies. Hudecek *et al*.^25^ found that ROR1 is highly expressed during early embryonic development but expressed at very low levels in adult tissues. Many papers reported that ROR1 has a very close relationship with chronic lymphocytic leukemia^26,27^ and acute lymphoblastic leukemia^28–32^. ROR1 is suggested as the targeted therapy for human malignancies^33,34^.
3. SNP rs4660992 maps gene BMP8B
  BMP8B is a thermogenic protein that increases brown adipose tissue thermogenesis through both central and peripheral actions and regulates the energy balance in partnership with hypothalamic AMPK^35^. Zhao *et al*.^36^ showed that mouse BMP8A (Op2) and BMP8B play roles in spermatogenesis and placental development. Ying *et al*.^37^ reported that BMP8B is required for the generation of primordial germ cells in mice.
4. SNP rs11766679 maps gene DPP6
  Genetic variation in DPP6 is associated with amyotrophic lateral sclerosis^20,38,39^ and familial idiopathic ventricular fibrillation^40^. Golz *et al*.^41^ found that DPP6 has been associated with a range of illnesses, including cancer, reproductive disorders, inflammation, and cardiovascular, endocrinological, metabolic, gastroenterological, hematological, muscle skeleton, neurological, urological, and respiratory diseases.
5. SNP rs12263497 maps gene INPP5F
  Zhu *et al*.^42^ reported that INPP5F is a polyphosphoinositide phosphatase that regulates cardiac hypertrophic responsiveness. Kim *et al*.^43^ found that INPP5F inhibits STAT3 activity and suppresses gliomas’ tumorigenicity. Palermo *et al*.^44^ reported that gene expression of INPP5F can be seen as an independent prognostic marker in fludarabine-based therapy of chronic lymphocytic leukemia. Bai *et al*.^45^ reported that alteration of the Akt signal plays an important role in diabetic cardiomyopathy. INPP5F is a negative regulator of Akt signaling.
6. SNP rs9263745 maps gene CCHCR1
  CCHCR1 is associated with malignant melanoma in the ClinVar database^15^. CCHCR1 is up-regulated in skin cancer and associated with EGFR expression^46^. The CCHCR1 (HCR) gene is relevant for skin steroidogenesis and downregulated in cultured psoriatic keratinocytes^47^.
7. SNP rs1552726 maps gene NLRP14
  NLRP14 may play a regulatory role in the innate immune system^48^. Mutations occur in the testis-specific NALP14 gene in men suffering from spermatogenic failure^49^. Westerveld *et al*.^14^ collected the data of 157 patients. They identified 25 suspicious variants: 1 nonsense mutation, 14 missense mutations, 6 silent mutations, and 4 intronic variants. By using ESEfinder and SpliceSiteFinder to check these SNPs, only the SNP rs1552726 was predicted to affect the correct splicing. Abe *et al*.^50^ reported that germ-cell-specific inflammasome component NLRP14 negatively regulates cytosolic nucleic acid sensing to promote fertilization.
8. SNP rs3742943 maps gene JAG2
  The gene ontology annotations related to JAG2 include Notch binding and calcium ion binding. The gene serves as a ligand for the Notch signaling receptors. The Notch signaling pathway is an intercellular signaling mechanism that is essential for proper embryonic development. Defects in JAG2 may cause ossifying fibroma and “shipyard eye”, according to the GeneCards database^49^. Houde *et al*.^51^ observed the overexpression of the NOTCH ligand JAG2 in malignant plasma cells from multiple myeloma patients and cell lines. Yustein *et al*.^52^ validated that the induction of ectopic Myc target gene JAG2 augments hypoxic growth and tumorigenesis in a human B-cell model. Asnaghi *et al*.^53^ reported that JAG2 promotes uveal melanoma dissemination and growth. Vaish *et al*.^54^ reported that JAG2 enhances tumorigenicity and chemoresistance of colorectal cancer cells.
9. SNP rs1646233 maps gene CBFA2T3
  CBFA2T3-GLIS2 is a fusion protein that defines an aggressive subtype of pediatric acute megakaryoblastic leukemia^55^. CBFA2T3-GLIS2 fusion transcript is a common feature in pediatric, cytogenetically normal AML, and it is not restricted to the FAB M7 subtype^56^. CBFA2T3-GLIS2-positive is closely related to pediatric non-Down syndrome acute megakaryoblastic leukemia^57^.
10. SNP rs11705619 maps gene TXNRD2
  Mutations in the gene TXNRD2 cause dilated cardiomyopathy^58^. Jakupoglu *et al*.^59^ investigated the Txnrd2 deletion and found that it leads to fatal dilated cardiomyopathy and morphological abnormalities of the cardiomyocytes. Prasad *et al*.^60^ reported that the TXNRD2 knockout is embryonically lethal in mice due to cardiac malformation.

A complete list of potentially lethal genes is in the Supplementary Material.

## Discussion

In this paper, we used the concepts learned from Mendel’s genetic experiments to propose a simple method that utilizes the distributions of genotypes among human populations to mine potentially lethal genes. Using the HapMap data, we selected 74 SNPs in 22 autosomal chromosomes, with 10 SNPs mapping “reviewed” genes in the NCBI database.

Among these genes, the LYST gene and ROR1 gene are related to fatal genetic childhood diseases. The genes JAG2, TXNRD2, and BMP8B play important roles in embryonic development and lead to many fatal diseases. The NALP14 gene may cause spermatogenic failure. Among the 25 variants, only SNP rs1552726 may affect the correct splicing of gene NLRP14^14^; rs1552726 is exactly 1 SNP within NLRP14, as detected by our method. The remaining genes, DPP6, INPP5F, CCHCR1, and CBFA2T3, are also associated with many genetic diseases. The results from our experiments were good and validated our approach. Our method can provide a narrow range of potentially lethal genes that deserve further study. More data will become available as whole-genome sequencing advances, so our method will become increasingly accurate. This method is a simple and inexpensive way to find the potentially pathogenic genes and SNPs.

## Methods

Given a bi-allele SNP, ‘A’ and ‘a’ are used to denote the major and minor allele, respectively. Since chromosomes come in pairs, each individual will take 1 of the following 3 genotypes: g0 = ‘AA’, g1 = ‘Aa’, or g2 = ‘aa’. In a population, the distribution of individuals taking each pattern can be counted. The abnormal distributions are the area of concern. Next, an example is given to illustrate the abnormal distributions.

Example 1: Suppose that there is a distribution for 1000 individuals; 500 individuals out of them take genotype ‘AA’ and other people take genotype ‘aa.’ Nobody takes the genotype ‘Aa.’

**Figure 1:**
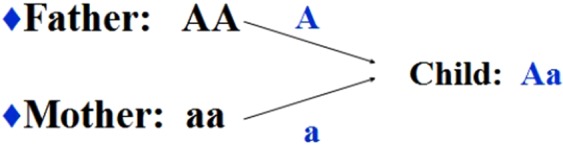
The heredity of SNPs.

According to the bisexual reproduction rule, a child will inherit 1 chromosome from his mother and 1 from his father. If the mother takes genotype ‘AA’ and the father takes genotype ‘aa’, the child will take genotype ‘Aa’. This example is shown in Fig. 1. Each woman has an equal probability to marry a man within this population. The probability of a child with genotype ‘Aa’ should be 2*0.5*0.5 = 0.5, which means there should be about 500 individuals taking the genotype ‘Aa.‘ However, none is observed. We think that the distribution on this SNP site is abnormal. The reason for the abnormal distribution is probably that most people taking the ‘Aa’ genotype will die in the embryonic state or die in childhood so that we cannot observe them as adults. We proposed the following hypothesis based on our analysis.

**Hypothesis 1:** Some genotypes should appear in human populations according to bisexual reproduction, but are not observed. These genotypes may cause gene abnormalities for fatal genetic diseases so that these fertilized embryos cannot finish their development or the carriers of these abnormalities die in childhood.

In the HapMap data, the SNP data of 11 human populations were sequenced. Since the relationships between the individuals in each population were unknown, we made an assumption to simplify the computation.

**Assumption 1:** In each population, every woman has the equal probability of marrying a certain man and giving birth to a baby.

For *population*_*j*_, the distribution *P* of individuals for all the genotypes can be counted. *P* = [*p*_0_, *p*_1_, *p*_2_], where *p*_*i*_ is the percentage of the individuals with genotype *g*_*i*_. Under Assumption 1 and the bisexual reproduction rule, the distribution among the next generation (denoted by P^*^) can be computed according to the distribution *P*. Let ${P}^{\ast }=[{p}_{0}^{\ast },{p}_{1}^{\ast },{p}_{2}^{\ast }]$. P^*^ can be computed by Formulas (1), (2) and (3).

1 $${p}_{0}^{\ast }={({p}_{0}+\frac{{p}_{1}}{2})}^{2},$$

2 $${p}_{1}^{\ast }=2({p}_{0}+\frac{{p}_{1}}{2})({p}_{2}+\frac{{p}_{1}}{2}),$$

and

3 $${p}_{2}^{\ast }={({p}_{2}+\frac{{p}_{1}}{2})}^{2}.$$

If there is no catastrophic event, the distribution among a human population will not change radically. Under normal circumstances, P^*^ can be treated as an approximation to the mean distribution of the current population. If *p*_*i*_ is 0, but ${p}_{i}^{\ast }$ is far from 0, then the distribution may be abnormal. Suppose the size of *population*_*j*_ is *n*_*j*_. The number of individuals matching the genotype *g*_*i*_ obeys the binomial distribution. *e*_*ij*_ is used to denote the event that *g*_*i*_ is not observed in the current *population*_*j*_, and the probability of *e*_*ij*_ is computed by Formula (4):

4 $${\Pr }({e}_{ij})={(1-{p}_{i}^{\ast })}^{{n}_{j}}.$$

In the HapMap data, there were 11 human populations. *eAll* denotes the event that the genotype *g*_*i*_ cannot be observed in all of the populations. The probability of *eAll* is given by Formula (5).

5 $$p\_value(eAll)=\prod _{j=0}^{10}Pr({e}_{ij}).$$

If *p*_*value*(*eAll*) is very small, the event when the genotype *g*_*i*_ is not observed in all of the populations is unlikely. However, it actually happened in our observation of the HapMap project. The reason may be that the genotype *g*_*i*_ can cause gene abnormalities for fatal genetic diseases and most of these affected people die in childhood. In a typical statistical test, 0.05 is often used as the threshold of the significance level. In our test, we needed to use a different approach since there were many SNPs to be checked. For example, there were 117068 SNPs on chromosome 1. The significance level needed to be corrected. The Bonferroni correction was used to adjust the threshold. Given *k* SNPs, 3 *k* hypotheses needed to be tested. The p-value threshold of significance was 0.05/3 *k*. The SNPs with *p*_*value*(*eAll*) below the p-value threshold were considered suspicious. After finding these SNPs, the potentially lethal genes were located in the NCBI database.

We assumed that every woman has the equal probability of marrying a certain man and giving birth to a baby in each population. This assumption simplified the computing. However, we actually should not exclude the possibility of further stratifications existing in each of the population groups. For the population with genotype g0 = ‘AA’ and genotype g1 = ‘Aa’, if genotype g2 = ‘aa’ is pathogenic, the number of false negatives will increase in the test because the stratification in the population will lead to an increase the percentage of people taking genotype g2 = ‘aa’ in the next generation. For the population with genotype g0 = ‘AA’ and genotype g2 = ‘aa’, if genotype g1 = ‘Aa’ is pathogenic, the number of false positives will increase in the test. However, we did not know the detailed stratifications in each population, so we used a strict Bonferroni correction to narrow the range of potentially lethal SNPs.

Our method is fully unsupervised. The software and codes are available at https://github.com/feathersky5000/ATest. Interested readers can download the HapMap^61^ data (genotypes data of the phase 3.3 consensus) and the software to replicate the results. The HapMap data contains 11 human populations, and 1417 individuals are sequenced. The details of the human populations are listed in Table 4.

**Table 4:** Information about the human populations used in this study.

Label	Population sample	Size
ASW	African Ancestry in the Southwest USA	87
CEU	Utah Residents with Northern and Western European	
	Ancestry from the CEPH Collection	174
CHB	Han Chinese in Beijing, China	139
CHD	Chinese in Metropolitan Denver, Colorado	109
GIH	Gujarati Indians in Houston, Texas	101
JPT	Japanese in Tokyo, Japan	116
LWK	Luhya in Webuye, Kenya	110
MEX	Mexican Ancestry in Los Angeles, California	86
MKK	Maasai in Kinyawa, Kenya	184
TSI	Toscans in Italy	102
YRI	Yoruba in Ibadan, Nigeria	209

## Supplementary information

sumplement
