# Supplementary material for: Locating potentially lethal genes using the abnormal distributions of genotypes
Source: Sci Rep. 2019 Jul 22;9:10543. doi: 10.1038/s41598-019-47076-w (PMC6646374; doi:10.1038/s41598-019-47076-w)
Supplement: Supplementary file 1 — sumplement [file 41598_2019_47076_MOESM1_ESM.pdf]

Locating potentially lethal genes using the abnormal distributions of genotypes

Xiaojun Ding[1], Xiaoshu Zhu[1*]

[1]School of Computer Science and Engineering, Yulin Normal University, Yulin, 537000, China

*ding.xiaojun@foxmail.com; xszhu@csu.edu.cn

A complete list of potentially lethal genes and SNPs

chr	SNP	GENE	RefSeq status	Alleles	diseasePattern	pvalue
1	rs11578666	LOC101060626	MODEL	C/T	CT	1.31E-08
1	rs1892438	OR2T10	PROVISIONAL	C/T	CC	4.51E-08
1	rs1977743	Unknown		C/T	CC	9.60E-09
1	rs2145402	LYST	REVIEWED	A/C	AA	3.05E-16
1	rs3134617	MYCL	VALIDATED	A/C	CC	1.31E-07
1	rs4660992	BMP8B	REVIEWED	C/T	TT	9.91E-10
1	rs4915931	ROR1	REVIEWED	A/G	AA	1.70E-12
1	rs6684448	DENND1B	VALIDATED	G/T	GG	2.74E-10
2	rs10171740	ANKRD36	PROVISIONAL	A/C	AA	1.56E-11
2	rs10874458	ANKRD36C	MODEL	A/C	CC	1.28E-09
2	rs1841377	Unknown		A/G	AG	1.04E-08
2	rs35614371	Unknown		A/C	CC	5.03E-10
2	rs698561	Unknown		G/T	GG	1.64E-16
2	rs790035	Unknown		A/C	CC	2.52E-14
3	rs2316811	Unknown		C/T	CC	3.85E-09
4	rs17777599	ZNF721	VALIDATED	A/G	AA	4.52E-12
4	rs1840589	Unknown		A/G	AG	1.08E-07
4	rs6855833	Unknown		G/T	GG	1.03E-09
6	rs11211686	Unknown		A/G	AA	4.48E-21
6	rs2926075	MEI4	INFERRED	A/G	AA	1.35E-26
6	rs4712994	BTN2A3P	VALIDATED	A/C	AA	1.78E-18
6	rs4713498	Unknown		G/T	TT	1.07E-09
6	rs6921855	Unknown		C/T	CC	3.84E-11
6	rs9263745	CCHCR1	VALIDATED	A/G	AA	5.67E-11
6	rs9277170	Unknown		A/G	GG	3.37E-18
7	rs11765720	Unknown		A/G	AA	3.69E-09
7	rs11766679	DPP6	REVIEWED	A/G	GG	4.29E-10
7	rs17132619	FAM185A	VALIDATED	A/G	GG	3.63E-16
7	rs17747927	LOC101929580	MODEL	G/T	GG	9.15E-09
7	rs1967430	Unknown		C/T	CC	6.47E-09
7	rs2533617	Unknown		A/G	AA	1.15E-20
7	rs34238522	LOC100133091	VALIDATED	C/T	CC	4.64E-18
7	rs4285375	Unknown		C/T	CC	1.29E-18
7	rs4721995	Unknown		G/T	GG	9.89E-10
7	rs6944297	ZNF138	VALIDATED	G/T	TT	1.62E-38
7	rs939760	Unknown		C/T	CC	1.04E-25
8	rs2945254	FAM86B3P	PROVISIONAL	G/T	TT	5.85E-23
8	rs3956240	Unknown		C/T	CC	8.23E-08
8	rs4875815	Unknown		C/T	CT	1.07E-07
8	rs7818545	Unknown		G/T	GT	1.45E-08
9	rs10985760	OR1L6	VALIDATED	C/T	TT	5.37E-12
9	rs3008128	Unknown		A/G	GG	8.93E-21
9	rs7467421	Unknown		C/T	CC	1.38E-24
10	rs12263497	INPP5F	REVIEWED	A/G	GG	1.46E-10
10	rs2559768	Unknown		G/T	GG	7.61E-14
11	rs1552726	NLRP14	REVIEWED	A/G	GG	2.64E-09
12	rs1057072	TUBA1B	VALIDATED	A/G	AG	1.78E-09
12	rs2536863	OVOS	MODEL	C/T	CT	1.36E-08
12	rs34598290	Unknown		C/T	TT	6.34E-11
12	rs584219	HCAR2	VALIDATED	C/T	CT	1.79E-09
12	rs7302302	Unknown		A/C	AA	1.51E-09
14	rs3742943	JAG2	REVIEWED	C/T	TT	6.07E-08
15	rs12101910	SCAPER	VALIDATED	G/T	GT	1.01E-07
15	rs12914262	Unknown		A/G	GG	1.45E-10

15	rs2254222	Unknown		G/T	GG	1.06E-07
15	rs28458204	GOLGA8N	VALIDATED	A/G	AG	1.36E-08
16	rs1646233	CBFA2T3	REVIEWED	A/G	AA	1.20E-09
16	rs17835978	Unknown		C/T	CC	2.69E-13
16	rs252283	Unknown		A/C	AA	2.41E-11
16	rs252304	Unknown		C/T	TT	1.60E-19
16	rs2547791	Unknown		G/T	GG	5.31E-14
16	rs2887622	Unknown		C/T	CC	1.73E-09
16	rs3743839	C16orf89	VALIDATED	A/G	AG	1.13E-07
16	rs7186433	ITFG1	PROVISIONAL	A/G	AG	1.00E-07
16	rs7196392	LOC102723773	MODEL	A/C	AA	2.02E-10
17	rs2107227	Unknown		C/T	CC	1.65E-09
17	rs2463524	Unknown		A/G	GG	2.10E-09
17	rs2532373	Unknown		C/T	CC	1.13E-09
17	rs2903882	PLEKHM1P	VALIDATED	G/T	GG	9.08E-15
17	rs3865264	ZNF286A	VALIDATED	G/T	GG	1.19E-12
19	rs2396225	CLEC17A	VALIDATED	G/T	GG	4.70E-13
20	rs6100383	Unknown		C/T	CT	1.48E-08
22	rs11089128	DUXAP8	VALIDATED	A/G	GG	2.35E-07
22	rs11705619	TXNRD2	REVIEWED	C/T	CC	1.43E-09
